# African Swine Fever Virus F317L Protein Inhibits NF-κB Activation To Evade Host Immune Response and Promote Viral Replication

**DOI:** 10.1128/mSphere.00658-21

**Published:** 2021-10-20

**Authors:** Jinping Yang, Shasha Li, Tao Feng, Xiangle Zhang, Fan Yang, Weijun Cao, Hongjun Chen, Huisheng Liu, Keshan Zhang, Zixiang Zhu, Haixue Zheng

**Affiliations:** a State Key Laboratory of Veterinary Etiological Biology, Lanzhou Veterinary Research Institute, Chinese Academy of Agricultural Sciences, Lanzhou, China; b National Foot and Mouth Diseases Reference Laboratory, Lanzhou Veterinary Research Institute, Chinese Academy of Agricultural Sciences, Lanzhou, China; c Key Laboratory of Animal Virology of Ministry of Agriculture, Lanzhou Veterinary Research Institute, Chinese Academy of Agricultural Sciences, Lanzhou, China; d Shanghai Veterinary Research Institute, Chinese Academy of Agricultural Sciences, Shanghai, China; Icahn School of Medicine at Mount Sinai

**Keywords:** ASFV, innate immune response, F317L, IκBα, NF-κB

## Abstract

African swine fever (ASF) is a highly contagious and deadly viral disease affecting pigs, with up to a 100% case fatality rate. The causative agent, African swine fever virus (ASFV), is a large multienveloped DNA virus which is the sole member of the family *Asfarviridae*. The double-stranded DNA genome of ASFV encodes more than 150 proteins; the functions of more than half of these viral proteins remain unknown. In this study, we determined that the uncharacterized protein F317L of ASFV had an antagonistic function against host innate immune response. F317L impaired NF-κB pathway activation by disruption of NF-κB activity. F317L interacted with IκB kinase β (IKKβ) and suppressed its phosphorylation, which subsequently reduced phosphorylation and ubiquitination of IκBα and enhanced IκBα stabilization. The accumulation of IκBα then blocked NF-κB activation and inhibited its nuclear translocation, resulting in decreased expression of various proinflammatory cytokines. As expected, overexpression of F317L promoted ASFV replication, and knockdown of F317L expression suppressed ASFV replication. This also indicated the crucial role of NF-κB pathway signaling in suppression of ASFV replication. Truncation mutation analysis indicated that the region spanning amino acids 109 to 208 of F317L was critical for inhibition of NF-κB activity. This is the first report about the function of F317L protein of ASFV, which provides insights for investigation of ASFV immune evasion mechanisms and development of ASFV live-attenuated vaccine.

**IMPORTANCE** African swine fever (ASF) is one of the most important pig diseases, causing a high case fatality rate and trade restrictions upon reported outbreaks. The limited understanding of the functions of the proteins of the causative agent, African swine fever virus (ASFV), has become a primary barrier to developing available commercial ASFV vaccines. ASFV infection causes severe immunosuppression. However, the mechanisms are still poorly understood. Identification of the viral factors responsible for causing immunosuppression will provide targets for developing ASFV live-attenuated vaccine through deletion of these viral factors. In this study, we determined that the uncharacterized protein F317L of ASFV had an antagonistic function against host innate immune response. Knockdown of F317L expression clearly inhibited ASFV replication. This is the first report about the function of F317L protein of ASFV, which provides new data to understand how ASFV inhibits host innate immune response and provides insights for developing ASFV live-attenuated vaccine.

## INTRODUCTION

Host antiviral immunity is divided into innate immunity and adaptive immunity. Innate immunity is the first line of defense for the host to recognize and resist virus infection (1). The innate immune response is induced in a rapid, nonspecific, universal, and diversified manner. Pattern recognition receptors (PRRs), as important components of innate immune system, recognize the elements of invading pathogens, such as the nucleic acids, proteins, sugars, and lipids of pathogens (2). The PRRs include RNA sensors, DNA sensors, and other sensors. The DNA sensors mainly recognize internalized extracellular cyclic dinucleotides, and the cyclic GMP-AMP synthase (cGAS) is currently considered the principal DNA sensor in various mammalian cells (3, 4), which recognizes various forms of cytoplasmic DNA coming from DNA viruses and intracellular parasites and other released DNA (5). The cGAS-STING (stimulator of interferon genes) signal pathway is the most classical pathway activated by DNA virus infection which is important for immune defense against the infection. After recognition of viral DNA, cGAS catalyzes the cyclization of ATP and GTP to cyclic guanosine-ADP (cGAMP) (6–9). As the second messenger, cGAMP activates STING on the endoplasmic reticulum (ER) surface (9–11), and the activated STING is transferred from the ER to the Golgi, forming a platform to activate transcription factors NF-κB and IRF3 via kinases IκB kinase (IKK) and TANK-binding kinase 1 (TBK1), respectively. IRF3 and NF-κB then enter the nucleus and induce the expression of interferons (IFNs) and other cytokines (12).

The NF-κB pathway is considered a prototypical proinflammatory signaling pathway which plays important roles in host innate immune response. NF-κB, as a transcriptional factor, is a critical regulator of the NF-κB pathway during DNA virus infection. In resting cells, NF-κB remains in the cytoplasm through its association with IκBα, and IκBα sequesters NF-κB by inhibiting its nuclear import. A critical step in activation of NF-κB is phosphorylation of IκBα by IKKs (13). The IKK complex has been suggested to be essential for activation of canonical NF-κB signaling in response to various stimuli, including cytokines (interleukin-1 [IL-1] and tumor necrosis factor alpha [TNF-α]), RNAs or DNAs derived from viruses, synthetic analogs, and other stimuli. Phosphorylation of IκBα results in its degradation through the ubiquitin-proteasome system, which subsequently leads to the release and nuclear translocation of NF-κB transcription factor (14).

African swine fever (ASF) is a severe, acute, highly contagious disease of pigs caused by African swine fever virus (ASFV). ASFV is the sole member of the family *Asfarviridae*, which infects domestic pigs and wild boars. ASFV infection causes lethargy, bloody diarrhea, vomiting, and high fever, with up to a 100% case fatality rate. The double-stranded DNA (dsDNA) genome of ASFV is around 170 to 193 kb and contains more than 150 open reading frames (ORF), encoding 150 to 200 proteins, with half having unknown functions (15). ASFV utilizes multiple mechanisms to inhibit and evade host immune response, which facilitates its replication. Currently, there is no available commercial vaccine against ASF (16). The innate immune pathways are crucial for the host to resist ASFV infection, while several ASFV proteins have been identified to block or disrupt host antiviral immune responses. A238L, MGF505, EP153R, and EP402R are involved in suppression of host innate immune response. A238L protein is an anti-inflammatory protein and has a structure similar to that of IκBα; it inhibits NF-κB activation and therefore efficiently suppresses host inflammatory response (17, 18). Multigene family proteins MGF505 and MGF360 inhibit host interferon production (19). CD2v lectin-like proteins encoded by EP402R and EP153R genes are involved in impairment of host defense system (20).

F317L protein is an uncharacterized protein of ASFV which consists of 317 amino acids (aa), and its function has remained unknown until now (21). In the present study, we determined that ASFV F317L is an inhibitor of the NF-κB pathway. F317L played an important role in suppression of NF-κB activation and production of proinflammatory cytokines, which, in turn, efficiently promoted ASFV replication.

## RESULTS

### F317L inhibited NF-κB promoter activation.

**FIG 1:**
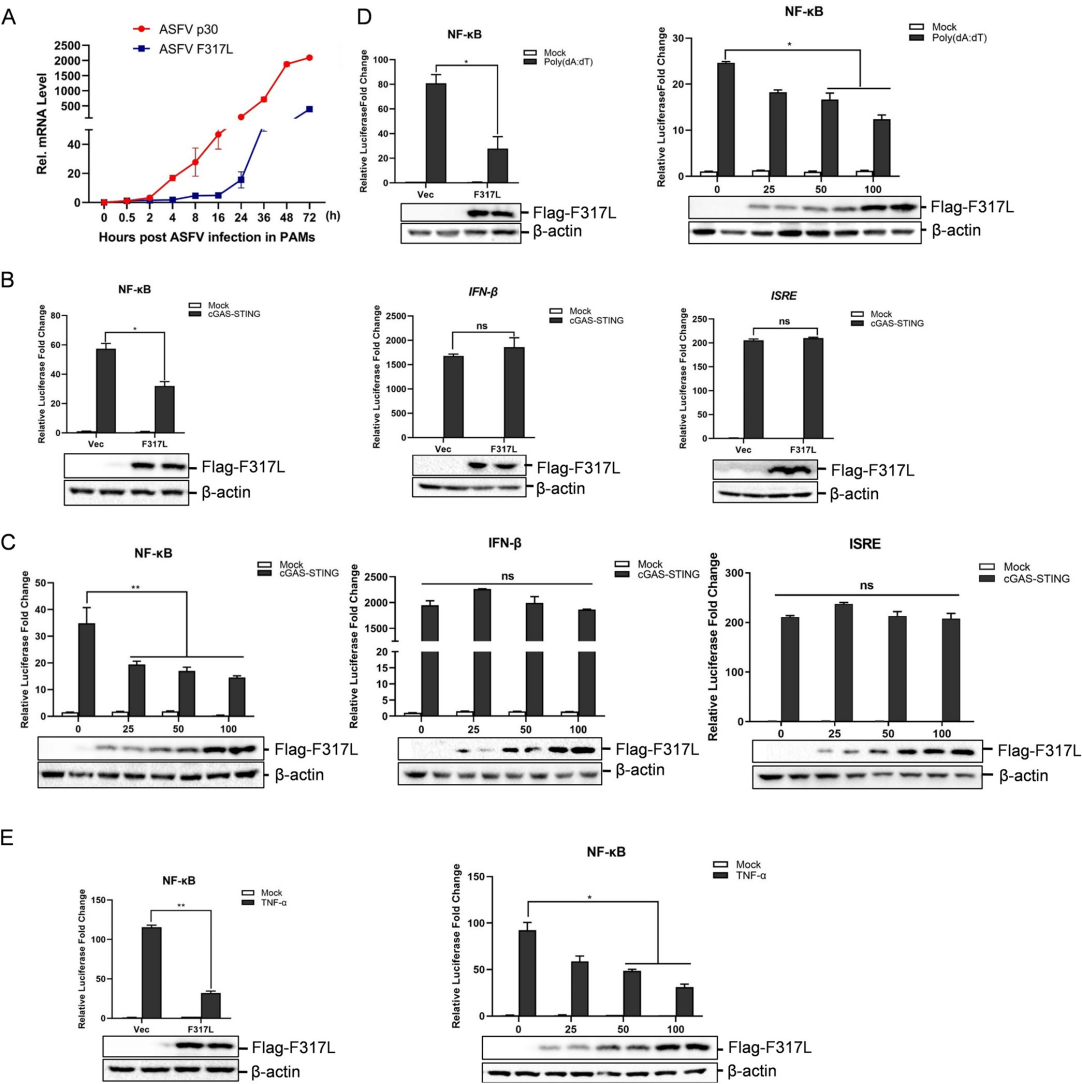
ASFV F317L inhibited cGAS-STING-, poly(dA-dT)-, and TNF-α-induced NF-κB promoter activation. (A) Primary porcine macrophages were infected by ASFV (MOI = 0.1) for the indicated time. The mRNA expression levels of ASFV F317L and p30 genes were measured by qPCR. (B) HEK-293T cells were cotransfected with vector or Flag-tagged F317L-expressing plasmids and NF-κB-luc, IFN-β-luc, or ISRE-luc reporter plasmids and pRL-TK internal control plasmids, along with vector (mock) or cGAS-STING-expressing plasmids. The luciferase activity was detected at 24 hpt. The expression of Flag-F317L protein was detected by Western blotting. (C) HEK-293T cells were cotransfected with vector or increasing amounts of Flag-tagged F317L-expressing plasmids and NF-κB-luc, IFN-β-luc, or ISRE-luc reporter plasmids and pRL-TK internal control plasmids, along with vector or cGAS-STING-expressing plasmids. The luciferase activity was detected at 24 hpt. The expression of Flag-F317L protein was detected by Western blotting. (D) HEK-293T cells were cotransfected with vector or Flag-tagged F317L-expressing plasmids and NF-κB-luc reporter plasmids and pRL-TK internal control plasmids for 24 h, followed by transfection with or without poly(dA-dT) for another 16 h. The luciferase activity was then measured. The right graph shows the dose-dependent experiment results. (E) HEK-293T cells were cotransfected with vector or Flag-tagged F317L-expressing plasmids and NF-κB-luc reporter plasmids and pRL-TK internal control plasmids for 24 h, followed by treatment with or without TNF-α (30 ng/ml) for another 8 h. The luciferase activity was then determined. The right graph shows the dose-dependent experiment results. *, *P < *0.05 (considered statistically significant); **, *P < *0.01 (considered highly significant).

The mRNA expression of F317L gene in ASFV-infected porcine primary macrophages (PAMs) was evaluated by quantitative PCR (qPCR) to confirm the viral replication and gene expression of F317L in PAMs. PAMs were infected with ASFV (multiplicity of infection [MOI] = 0.1) for 0, 0.5, 2, 4, 8, 16, 24, 36, 48, or 72 h. The expression of a well-characterized gene, the ASFV p30 gene, was used as a positive control. The expression pattern of p30 was consistent with that described in a previous report (22) (Fig. 1A). High levels of F317L mRNA were detected after 4 h of infection and then increased quickly as infection progressed (Fig. 1A). This confirmed the expression of F317L in ASFV-infected cells. cGAS-STING pathway activation is crucial for initiation of host antiviral response by induction of IFNs and proinflammatory cytokine production during DNA virus infection (13). To explore whether F317L is involved in regulation of host innate immune response, cGAS-STING-induced IFN-β, NF-κB, and interferon-sensitive response element (ISRE) promoter activation was evaluated in F317L-overexpressing cells. Overexpression of F317L clearly suppressed cGAS-STING-induced NF-κB promoter activation but not IFN-β or ISRE promoter activation (Fig. 1B). Dose-dependent experiments were performed which confirmed the inhibitory effect of F317L on NF-κB promoter activation (Fig. 1C). To firmly determine the inhibitory effect of F317L on NF-κB promoter activation, poly(dA-dT)- and TNF-α-induced NF-κB promoter activation was evaluated. Overexpression of ASFV F317L protein remarkably inhibited poly(dA-dT)-induced (Fig. 1D) and TNF-α-induced (Fig. 1E) NF-κB promoter activation as well, showing a dose-dependent manner. These data indicated that F317L suppressed NF-κB promoter activation.

### F317L inhibited TNF-α and poly(dA-dT)-induced proinflammatory cytokine expression.

**FIG 2:**
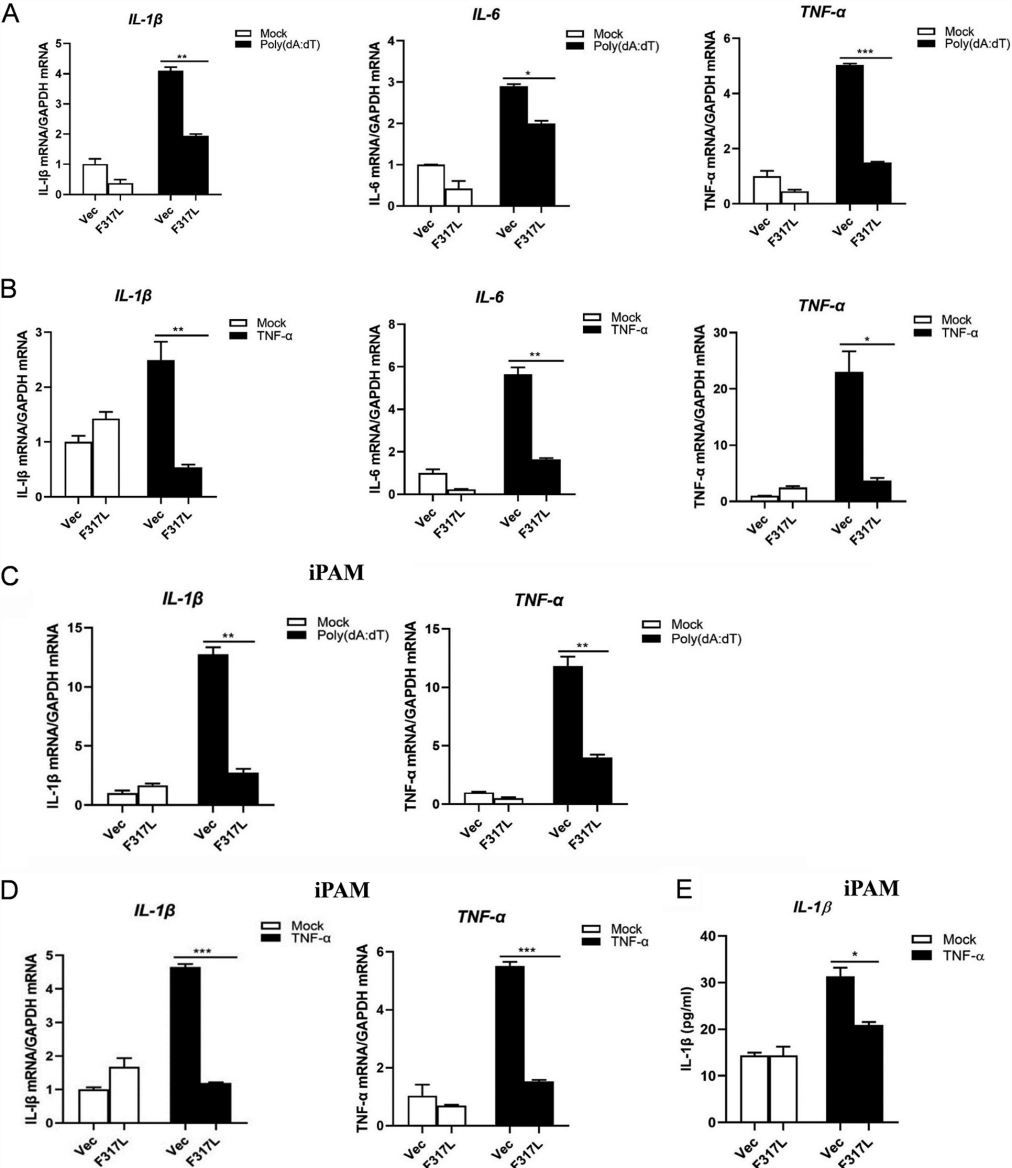
F317L suppressed proinflammatory cytokine expression induced by poly(dA-dT) and TNF-α. (A) HEK-293T cells were transfected with empty vector or F317L-expressing plasmid for 24 h and then mock transfected or transfected with poly(dA-dT) for another 16 h. The mRNA expression level of IL-1β, IL-6, and TNF-α was determined by qPCR. (B) HEK-293T cells were transfected with empty vector or F317L-expressing plasmid for 24 h and then mock treated or treated with TNF-α for 8 h. The mRNA expression level of IL-1β, IL-6, and TNF-α was measured by qPCR. (C) iPAM cells were transfected with empty vector or F317L-expressing plasmid for 24 h and then mock transfected or transfected with poly(dA-dT) for another 16 h. The mRNA expression level of IL-1β and TNF-α was determined by qPCR. (D and E) iPAM cells were transfected with empty vector or F317L-expressing plasmid for 24 h and then mock treated or treated with TNF-α (30 ng/ml) for 8 h. The mRNA expression level of IL-1β and TNF-α was measured by qPCR (D). The secretion of IL-1β in the cell culture supernatant was detected by ELISA (E).

cGAS-STING- and TNF-α-induced NF-κB activation contributes to the expression of a variety of proinflammatory cytokines, including IL-1β, TNF-α, and IL-6 during DNA virus infection (23). F317L inhibited the activation of NF-κB promoter activation; we thus examined whether F317L inhibited the expression of proinflammatory cytokines. Poly(dA-dT)-induced mRNA expression levels of IL-1β, IL-6, and TNF-α were evaluated in F317L-overexpressing cells. Transfection of poly(dA-dT) efficiently induced expression of IL-1β, IL-6, and TNF-α. However, overexpression of F317L considerably inhibited poly(dA-dT)-induced expression of these proinflammatory cytokines (Fig. 2A). Meanwhile, we evaluated the effect of F317L on TNF-α-induced mRNA expression levels of IL-1β, IL-6, and TNF-α. As expected, overexpression of F317L considerably inhibited TNF-α-induced expression of these proinflammatory cytokines (Fig. 2B). This inhibitory effect was also confirmed in a porcine macrophage line (iPAM cells), which showed that F317L suppressed proinflammatory cytokine expression in iPAM cells as well (Fig. 2C and D). The secretion of IL-1β in iPAM cell culture supernatants was further detected, and the trajectory was consistent with the qPCR results; TNF-α-induced secretion of IL-1β was considerably decreased in the presence of F317L protein (Fig. 2E). Together, these data suggested that F317L restrained the expression of proinflammatory cytokines.

### Effect of ASFV infection on proinflammatory cytokine expression.

**FIG 3:**
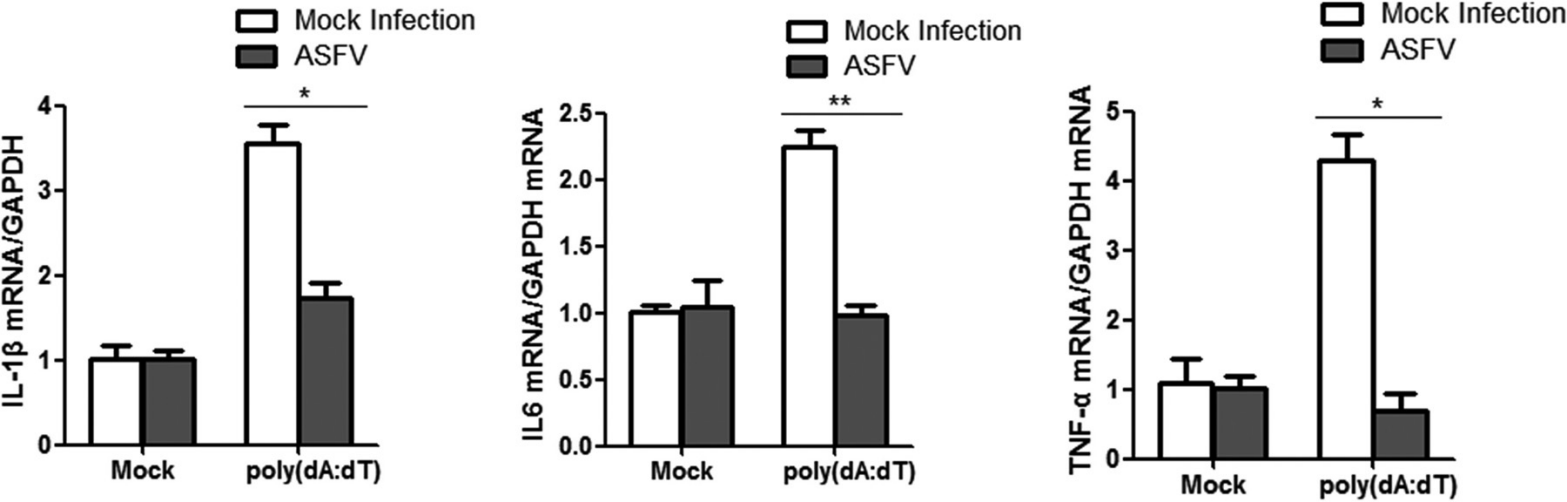
ASFV infection suppressed IL-1β, IL-6, and TNF-α expression. iPAM cells were mock treated or transfected with poly(dA-dT) for 4 h. The poly(dA-dT)-transfected cells were subsequently mock infected or infected by ASFV (MOI, 0.01) for 24 h. All the cells were then collected, and the mRNA levels of IL-1β, IL-6, and TNF-α were measured by qPCR assay.

To evaluate the influence of ASFV infection on expression of proinflammatory cytokines, iPAM cells were mock treated, transfected with poly(dA-dT), or transfected with poly(dA-dT) followed by ASFV infection for 24 h. The mRNA expression levels of IL-1β, IL-6, and TNF-α were then measured by qPCR. The results indicated that ASFV infection significantly decreased poly(dA-dT)-induced IL-1β, IL-6, and TNF-α mRNA expression (Fig. 3). This suggested that ASFV infection inhibited proinflammatory cytokine expression and that F317L was involved in this antagonizing effect.

### Overexpression of F317L protein promoted ASFV replication, and knockdown of F317L inhibited ASFV replication.

**FIG 4:**
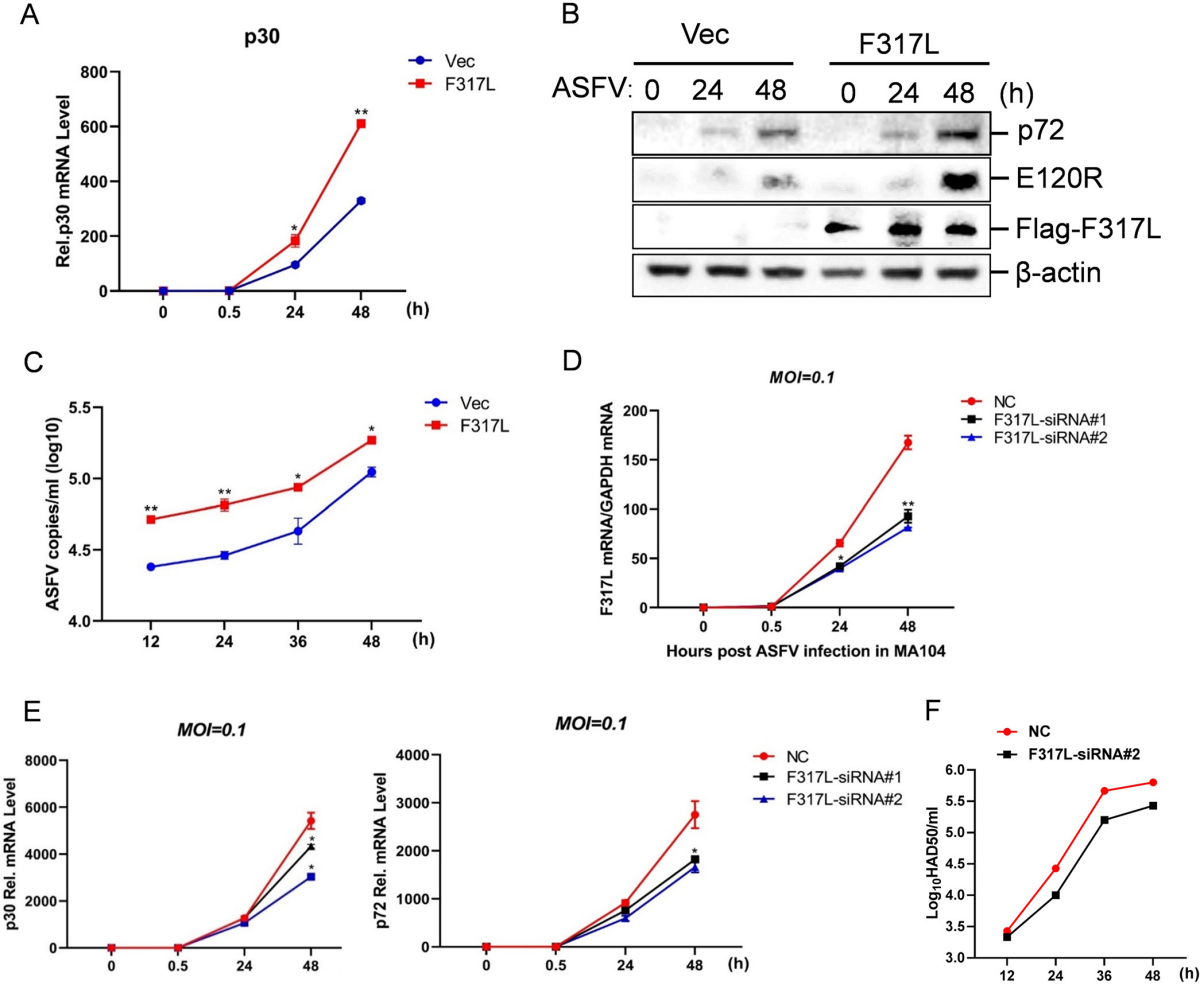
ASFV F317L protein was essential for ASFV replication. (A) MA104 cells were transfected with F317L-expressing plasmid or empty vector for 24 h, followed by ASFV infection for 0, 0.5, 24, or 48 h. The expression level of viral p30 mRNA was detected by qPCR. (B) MA104 cells were transfected with F317L-expressing plasmids or empty vector for 24 h, followed by ASFV infection for 0, 24, or 48 h, and the expression of Flag-F317L, p72, and E120R protein was detected by Western blotting. (C) MA104 cells were transfected with F317L-expressing plasmid or empty vector for 24 h, followed by ASFV infection for 12, 24, 36 or 48 h. ASFV copies in cell supernatant were detected by qPCR using TaqMan DNA probes. (D) MA104 cells were transfected with NC siRNA, F317L siRNA1, or F317L siRNA2, and at 6 hpt, the cells were infected with ASFV for 0, 24, or 48 h. mRNA expression of F317L was detected by qPCR to confirm the efficacy of the transfected siRNAs. (E) MA104 cells were transfected with NC siRNA, F317L siRNA1, or F317L siRNA2. At 6 hpt, the cells were infected with ASFV for 0, 0.5, 24, or 48 h, and the mRNA expression levels of viral p30 and p72 were detected by qPCR. (F) MA104 cells were transfected with NC siRNA or F317L siRNA2. At 6 hpt, the cells were infected with ASFV for 12, 24, or 48 h. The virus yields at different times postinfection were expressed as log_10_ HAD_50_ per milliliter. HAD_50_ represents 50% hemadsorbing doses.

Since ASFV F317L antagonized the cGAS-STING-mediated antiviral response, we further investigated its regulatory effect on ASFV replication. The p30 mRNA level and p72 and E120R protein expression levels were used as the replicative indicator of ASFV. Overexpression of F317L markedly enhanced ASFV replication at 24 and 48 h postinfection (hpi) compared with that in the vector-transfected cells (Fig. 4A and B). Similarly, the viral RNA amounts in the supernatant were evaluated by the TaqMan qPCR, which confirmed that overexpression of F317L promoted ASFV replication (Fig. 4C). Meanwhile, we investigated the state of ASFV replication in F317L small interfering RNA (siRNA) cells as well. Two F317L siRNAs that specifically targeted F317L were designed and synthesized, and the knockdown efficiency was confirmed by qPCR (Fig. 4D). MA1104 cells were transfected with negative control (NC) siRNA, F317L siRNA1, or F317L-siRNA2, the cells were infected with ASFV at 6 h posttransfection (hpt), and the viral replication level was evaluated at 0, 0.5, 24, and 48 hpi. The p30 and p72 mRNA expression levels were used as the replicative indicator of ASFV. Knockdown of F317L clearly decreased ASFV replication and virus yields (Fig. 4E and F). These results suggested that F317L played a positive regulatory role in ASFV replication and was essential for ASFV replication.

### F317L protein interacted with the adaptor protein IKKβ in the cGAS-STING pathway.

**FIG 5:**
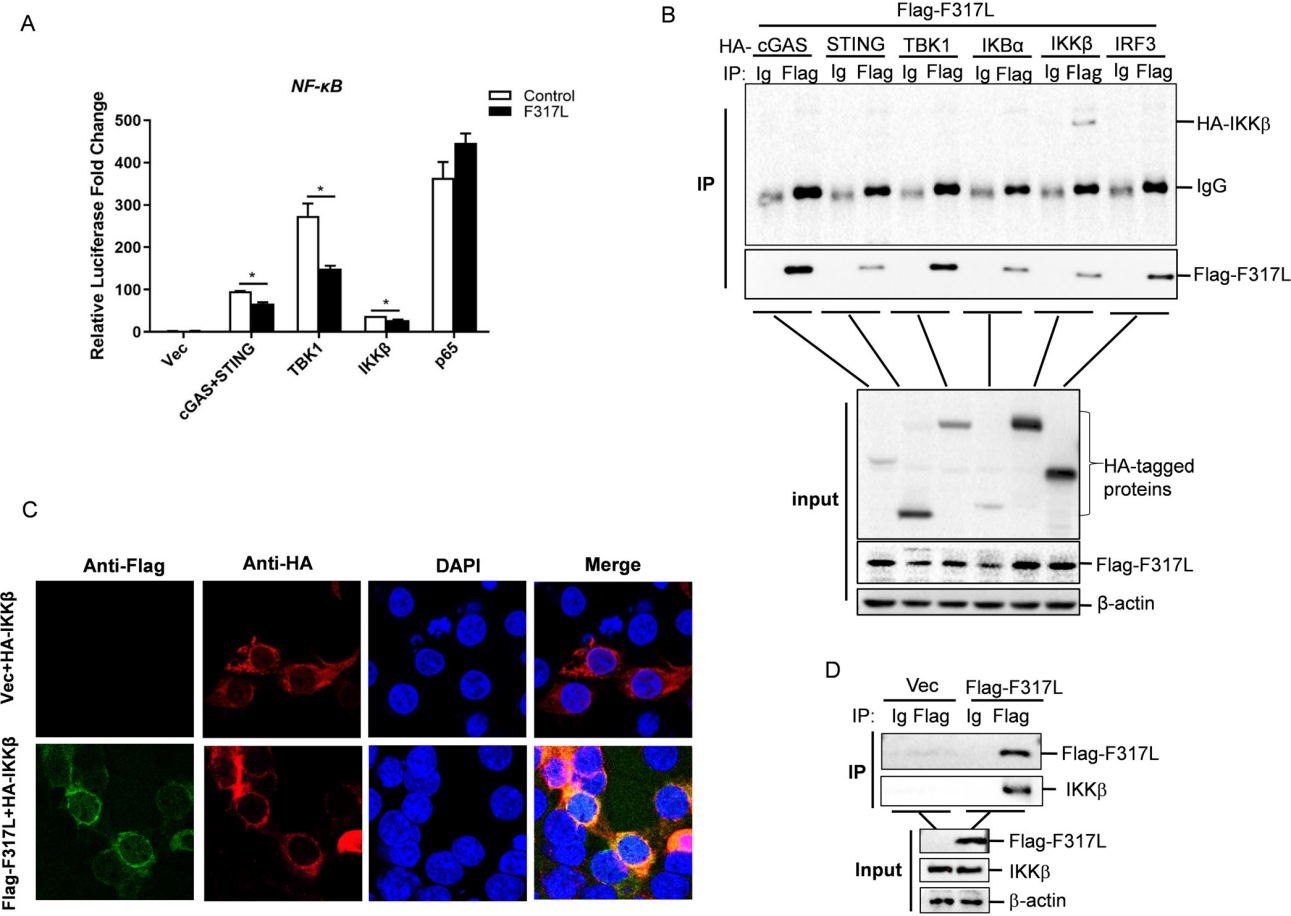
F317L potentially targeted IKKβ to impair NF-κB pathway activation. (A) HEK-293T cells were cotransfected with empty vector or F317L-expressing plasmids, and vector or the indicated plasmid expressing cGAS-STING, TBK1, IKKβ, or p65, together with NF-κB-luc and pRL-TK plasmids. The luciferase activity was measured at 24 hpt. (B) HEK-293T cells were cotransfected with empty vector or F317L-expressing plasmids and the indicated plasmids expressing HA-tagged cGAS, STING, TBK1, IκBα, IKKβ, or p65 for 36 h. The cells were then lysed and immunoprecipitated by anti-Flag and anti-IgG antibody. The whole-cell lysates (input) and immunoprecipitation (IP) complexes were analyzed by Western blotting using anti-HA, anti-Flag, or anti-β-actin antibodies. (C) HEK-293T cells were cotransfected with empty vector or Flag-F317L-expressing plasmid and HA-IKKβ-expressing plasmid for 24 h. The subcellular localization of IKKβ and F317L protein was then analyzed by IFA. (D) HEK-293T cells were transfected with empty vector or F317L-expressing plasmids for 36 h. The cells were lysed and immunoprecipitated by anti-Flag and anti-IgG antibody. The whole-cell lysates (input) and IP complexes were analyzed by Western blotting using anti-Flag, anti-IKKβ, or anti-β-actin antibodies.

In the cGAS-STING pathway, STING activates NF-κB by both canonical and noncanonical manners, and several components are involved in this process (13, 24). The observed blockade of NF-κB activation by F317L protein raised the possibility that F317L protein targeted one or several components of the cGAS-STING pathway. To investigate which component might be a potential target of F317L, the effect of F317L on cGAS-STING-, TBK1-, IKKβ-, or p65-induced NF-κB promoter activation was evaluated. The results showed that F317L significantly blocked cGAS-STING-, TBK1-, and IKKβ-induced NF-κB promoter activation. However, it did not affect p65-induced NF-κB promoter activation (Fig. 5A). IKKβ is an upstream molecule of p65 which is critical for activation of NF-κB by both TNF-α and dsDNA. Therefore, we speculated that ASFV F317L protein might target IKKβ to block NF-κB activation and suppress proinflammatory cytokine expression. The interaction between cGAS, STING, TBK1, IKKβ or p65, and F317L was also investigated by performing a coimmunoprecipitation (co-IP) experiment, which showed that F317L interacted with IKKβ (Fig. 5B). The colocalization of F317L and IKKβ was also evaluated. F317L indeed colocalized with IKKβ in the cytoplasm (Fig. 5C). The interaction between F317L and endogenous IKKβ was further evaluated, and the results showed that F317L interacted with endogenous IKKβ (Fig. 5D). This implied that F317L interacted with IKKβ and blocked NF-κB activation.

### ASFV F317L protein inhibited IKKβ phosphorylation, which, in turn, decreased IκBα phosphorylation and upregulated IκBα expression.

**FIG 6:**
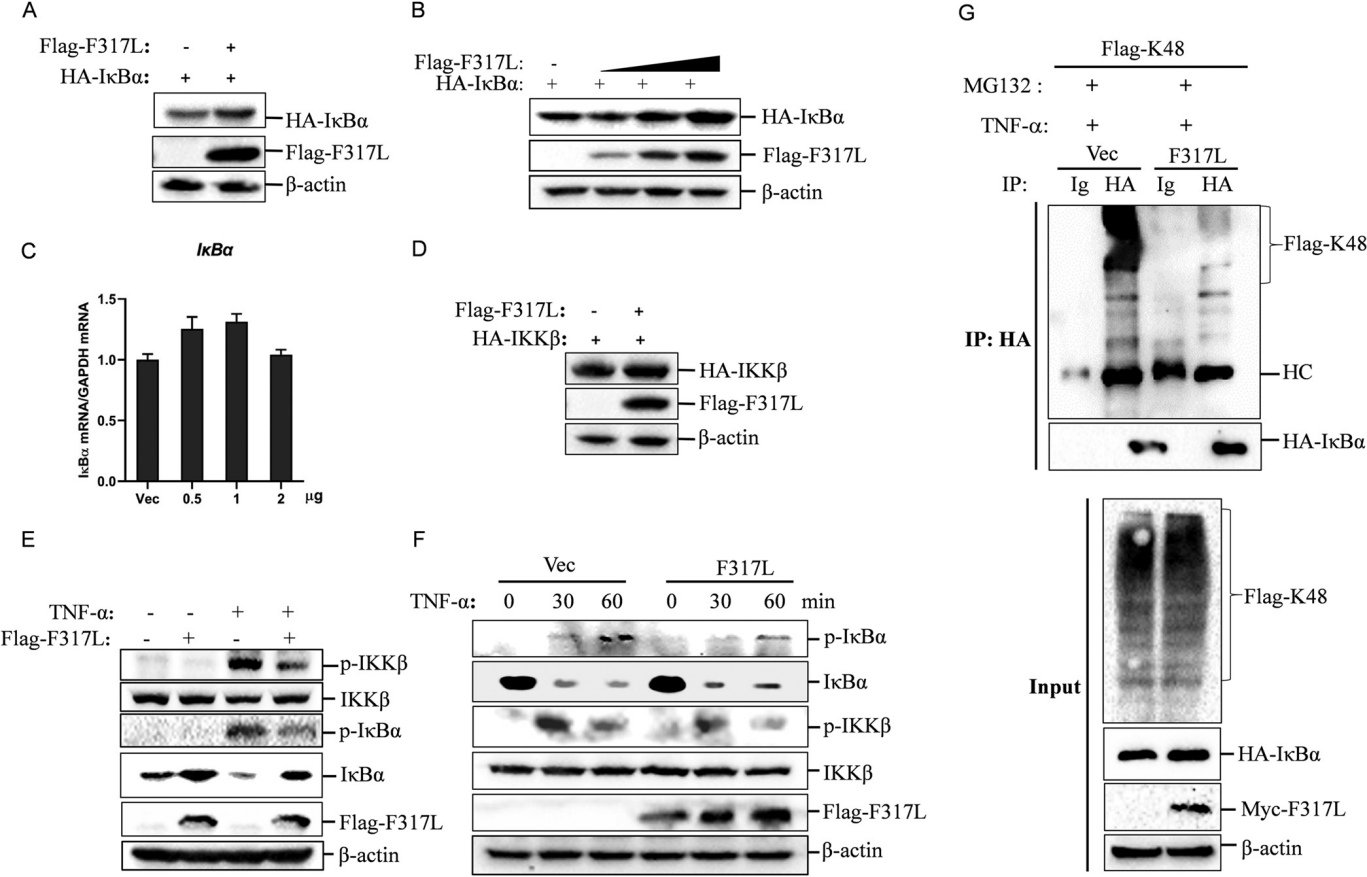
ASFV F317L inhibited phosphorylation of IKKβ to increase the abundance of IκBα. (A) HEK-293T cells were cotransfected with empty vector or Flag-F317L-expressing plasmids and HA-IκBα-expressing plasmids for 24 h. The expression of HA-IκBα and Flag-F317L was detected by Western blotting. (B and C) HEK-293T cells were cotransfected with empty vector or increasing amounts of Flag-F317L-expressing plasmids and HA-IκBα-expressing plasmids for 24 h. The protein expression of HA-IκBα and Flag-F317L was detected by Western blotting (B), and the relative expression of IκBα mRNA was determined by qPCR (C). (D) HEK-293T cells were cotransfected with empty vector or Flag-F317L-expressing plasmids and HA-IKKβ-expressing plasmids for 24 h. The expression of HA-IKKβ and Flag-F317L was detected by Western blotting. (E) HEK-293T cells were transfected with empty vector or F317L-expressing plasmids for 24 h, and the cells were then mock treated or treated with TNF-α for 1 h. The expression of IKKβ, phosphorylated IKKβ (p-IKKβ), IκBα, phosphorylated IκBα (p-IκBα), and F317L protein was detected by Western blotting. (F) HEK-293T cells were transfected with empty vector or F317L-expressing plasmids for 24 h, and the cells were then mock treated or treated with TNF-α for 0, 0.5, or 1 h. The expression of IKKβ, p-IKKβ, IκBα, p-IκBα, and F317L protein was detected by Western blotting. (G) HEK-293T cells were cotransfected with Myc empty vector or Myc-F317L-expressing plasmids and HA-IκBα as well as Flag-K48 ubiquitin plasmids for 30 h. The cells were then treated with TNF-α in the presence of MG132 for 6 h. The cell lysates were then immunoprecipitated by anti-HA and anti-IgG antibody. The whole-cell lysates (input) and IP complexes were analyzed by Western blotting using anti-HA, anti-Flag, anti-Myc, or anti-β-actin antibodies.

In resting cells, NF-κB is sequestered in the cytoplasm through association with IκBα (inhibitor of NF-κB) (25). A crucial step in activation of NF-κB is phosphorylation of IκBα by phosphorylated IKKβ to cause the degradation of IκBα through the proteasome (13). Therefore, the ubiquitination and degradation of IκBα are critical for NF-κB activation (26). The expression of hemagglutinin (HA)-tagged IκBα in F317L-overexpressing cells was detected by Western blotting, which revealed that overexpression of F317L clearly enhanced the expression of IκBα (Fig. 6A). The dose-dependent experiment further confirmed that F317L stabilized the expression of IκBα in a dose-dependent manner (Fig. 6B). Meanwhile, we determined that overexpression of F317L did not affect the mRNA expression of IκBα (Fig. 6C). In addition, F317L did not affect the expression of IKKβ (Fig. 6D). F317L interacted with IKKβ, and we speculated that F317L suppressed the activation of IKKβ, resulting in the accumulation of IκBα and inhibition of NF-κB activation. Therefore, we investigated whether F317L protein affected the phosphorylation of IKKβ and IκBα. We found that F317L protein indeed blocked TNF-α-induced phosphorylation of IKKβ and IκBα (Fig. 6E). The time course experiment further confirmed this regulatory effect (Fig. 6F). The ubiquitination of IκBα was further detected, and we found that F317L protein indeed blocked TNF-α-induced ubiquitination of IκBα (Fig. 6G). These data indicated that F317L interacted with IKKβ and suppressed phosphorylation of IKKβ, which subsequently reduced phosphorylation as well as ubiquitination of IκBα and led to the increased abundance of IκBα and suppression of NF-κB activation.

### ASFV F317L protein inhibited p65 nuclear translocation.

**FIG 7:**
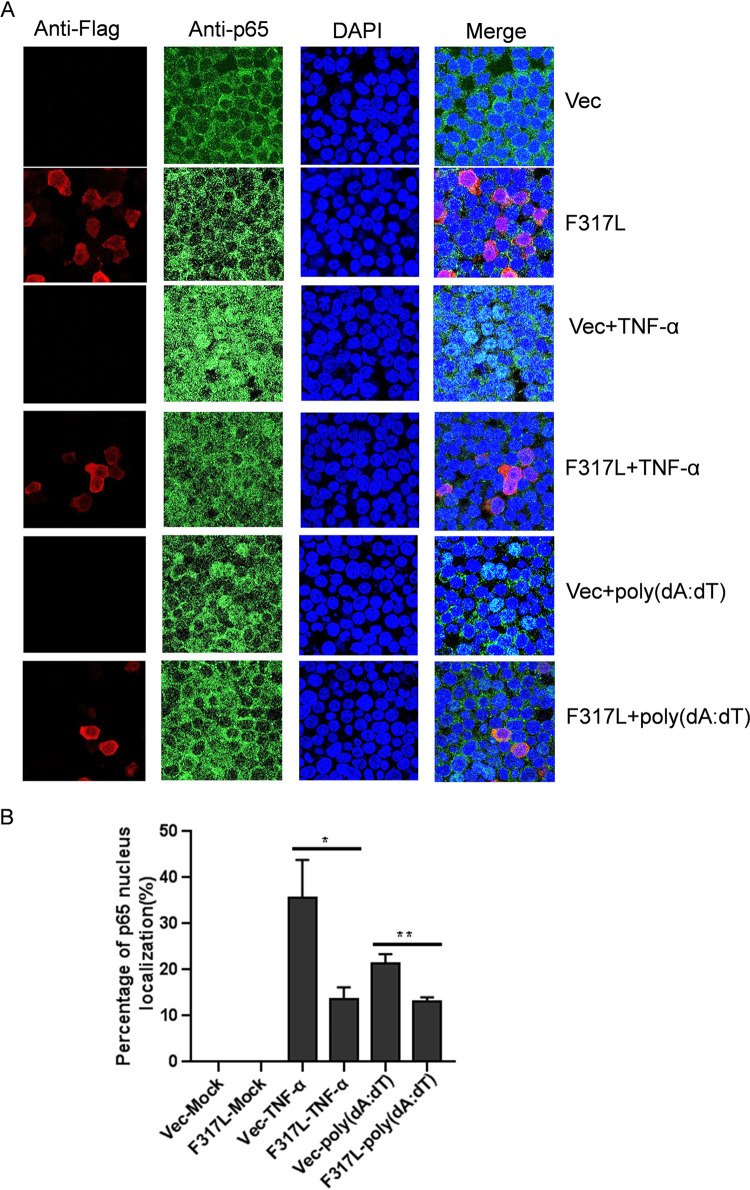
ASFV F317L inhibited nuclear translocation of p65. (A) HEK-293T cells were transfected with empty vector or Flag-F317L-expressing plasmids for 24 h, and the transfected cells were treated with 30 ng/ml of TNF-α for 30 min or transfected with 1 μg of poly(dA-dT) for 12 h. The cells were then fixed and subjected to IFA analysis. Red represents F317L, green represents p65, and blue indicates nuclei. (B) Statistical analysis of p65 nuclear translocation in ASFV F317L-overexpressing cells was carried out. One hundred cells were counted in the randomly selected visual fields three times, and p65 nuclear localization ratio was evaluated.

p65 nuclear translocation is the indicator of NF-κB activation, and its nuclear translocation requires the protein being released from the p65/IκB complex. We have determined that F317L stabilized IκBα abundance and blocked NF-κB promoter activation and proinflammatory cytokine expression. To evaluate the state of p65 nuclear translocation in the presence of F317L protein, TNF-α- and poly(dA-dT)-induced nuclear translocation of p65 in F317L protein-expressing cells was evaluated by performing indirect immunofluorescence assay (IFA). TNF-α treatment and poly(dA-dT) transfection clearly induced nuclear translocation of p65 (Fig. 7A). However, overexpression of F317L remarkably decreased both TNF-α- and poly(dA-dT)-induced p65 nuclear translocation (Fig. 7A). The statistical analysis confirmed that overexpression of F317L protein inhibited p65 nuclear translocation (Fig. 7B). This firmly suggested that F317L inhibited NF-κB activation.

### The region from amino acids 108 to 209 of F317L was essential for suppression of NF-κB activation.

**FIG 8:**
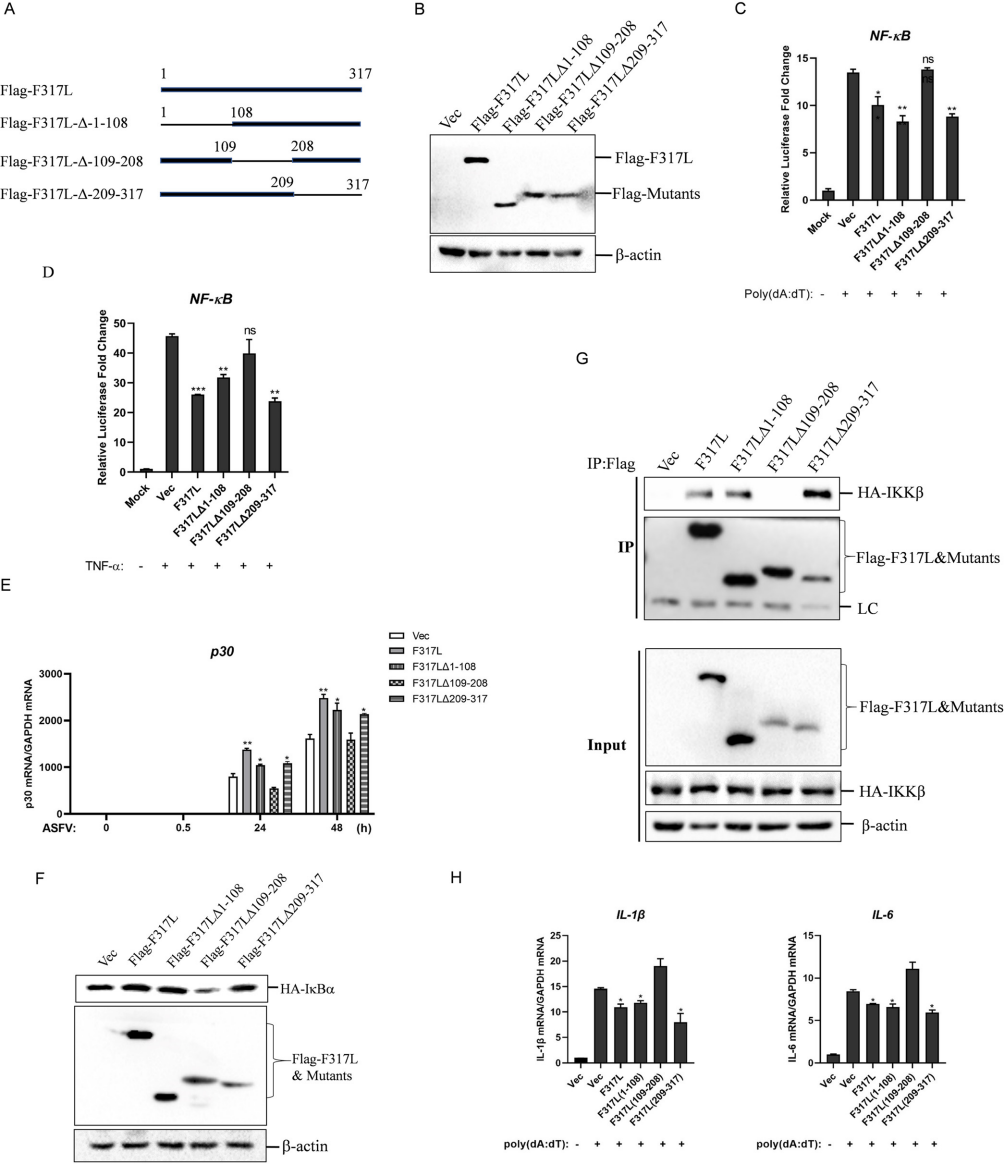
Deletion of the region from amino acids 108 to 209 in F317L abrogated F317L-mediated antagonistic function against NF-κB. (A) Schematic representation of truncation mutants of F317L. (B) HEK-293T cells were transfected with the constructed mutant-expressing plasmids for 24 h, and the expression of the indicated mutants was detected by Western blotting. (C) HEK-293T cells were cotransfected with vector, F317L, or the indicated truncated mutant-expressing plasmids, together with NF-κB-Luc reporter plasmids and pRL-TK plasmids for 24 h, followed by transfection with or without poly(dA-dT) for another 12 h. The luciferase activity was then measured. (D) HEK-293T cells were cotransfected with vector, F317L, or the indicated truncated mutant-expressing plasmids, together with NF-κB-Luc reporter plasmids and pRL-TK plasmids for 24 h; the cells were then treated or not with 30 ng/ml of TNF-α for 8 h. The luciferase activity was then determined. (E) MA104 cells were transfected with vector, F317L, or the indicated truncated mutant-expressing plasmids for 24 h, and the cells were infected by ASFV for 0, 0.5, 24, or 48 h. The viral p30 mRNA level was then detected by qPCR. (F) HEK-293T cells were cotransfected with vector, Flag-F317L, or the indicated truncated mutant-expressing plasmids and HA-IκBα-expressing plasmids for 24 h, and the protein expression levels of HA-IκBα, Flag-F317L, and truncated mutants were detected by Western blotting. (G) HEK-293T cells were cotransfected with empty vector, F317L, or the indicated truncated mutant-expressing plasmids and HA-IKKβ-expressing plasmids for 36 h. The cells were then lysed and immunoprecipitated by anti-Flag antibody. The IP and whole-cell lysate (input) complexes were analyzed by Western blotting using anti-HA, anti-Flag, or anti-β-actin antibodies. (H) HEK-293T cells were transfected with vector, F317L, or the indicated truncated mutant-expressing plasmids for 24 h, followed by transfection with or without poly(dA-dT) for another 16 h. The mRNA expression levels of IL-1β and IL-6 were then measured by qPCR.

To identify the crucial region in F317L responsible for suppression of NF-κB activation, we generated three plasmids expressing F317L truncated mutants (Fig. 8A). The expression of the truncated mutants was confirmed by Western blotting (Fig. 8B). The effect of these mutants on poly(dA-dT)- and TNF-α-induced activation of the NF-κB promoter was then evaluated. Overexpression of F317L, F317LΔ1-108, and F317LΔ209-317 but not F317LΔ109-208 inhibited poly(dA-dT)- and TNF-α-triggered NF-κB promoter activation (Fig. 8C and D). Meanwhile, we found that overexpression of F317L, F317LΔ1-108, and F317LΔ209-317 but not F317LΔ109-208 increased ASFV replication (Fig. 8E). The effect of these mutants on expression of IκBα was evaluated as well. As expected, F317LΔ109-208 failed to increase IκBα expression (Fig. 8F). The interaction between F317LΔ109-208 and IKKβ was evaluated subsequently, and the results showed that F317LΔ109-208 failed to bind to IKKβ (Fig. 8G). In addition, we found that overexpression of F317LΔ109-208 could not inhibit poly(dA-dT)-induced IL-1β or IL-6 mRNA expression (Fig. 8H). Collectively, these data suggested that the region from aa 109 to 208 in F317L was critical for suppression of NF-κB activation through interaction with IKKβ.

## DISCUSSION

The innate immune system is the first line of host defense against viral infection (27). The cGAS-STING pathway plays an important role in DNA virus infection by initiating type I IFN signaling (STAT1 axis) and NF-κB signaling. STING activates canonical and noncanonical NF-κB activation via the TNF receptor-associated factor 6 (TRAF6)-TBK1 axis and TRAF3, respectively (13). TNF-α is one of the most potent physiological inducers of NF-κB as well. Although STING activation leads to multiple outcomes, such as type Ι IFN production, proinflammatory cytokine production, and autophagy, most studies on the cGAS-STING pathway were focused on type Ι IFN production (28). Early induction of proinflammatory cytokines by NF-κB is critical for inhibition of replication of various viruses. NF-κB plays an important role in regulation of proinflammatory cytokine expression, which helps mount the inflammatory response and recruit immune cells to the site of infection. Various viruses have evolved multiple strategies to counteract NF-κB function.

ASFV infection suppresses NF-κB pathway activation. ASFV I329L targets the TRIF protein to antagonize TLR3-mediated innate immunity and interfere with NF-κB and IRF3 activation (29). ASFV DP96R inhibits TBK1 phosphorylation to block type I IFN production and NF-κB activation (30). ASFV A238L contains ankyrin repeats homologous to the ankyrin repeats of IκB, and it interacts with p65 of the NF-κB complex, thereby inhibiting NF-κB activation (17, 18, 31). Many other viruses have also evolved complex countermeasures to subvert the innate immune response; for example, vaccinia virus protein B14 binds to IKKβ to prevent IKKβ autophosphorylation and activation, thereby inhibiting NF-κB signaling (32). Hepatitis C virus (HCV) nonstructural protein NS3 competes with IKKγ to bind with a ubiquitin ligase LUBAC, which results in decreased LUBAC-mediated linear ubiquitination of IKKγ, thus inhibiting the activation of NF-κB (33). The porcine epidemic diarrhea virus (PEDV) NSP1 inhibits the phosphorylation and degradation of IκBα and therefore blocks p65 nuclear translocation and inhibits the activation of NF-κB (34).

Our results revealed for the first time that F317L significantly antagonizes cGAS-STING/poly(dA-dT)/TNF-α-mediated activation of NF-κB. Meanwhile, we found that F317L did not inhibit cGAS-STING-induced type I IFN production or the type I IFN response (Fig. 1), suggesting that F317L specifically inhibits NF-κB activation and does not affect the immune sensors and adaptors in the cGAS-STING pathway shared by IRF3 and NF-κB. However, it targets the component shared by the TNF-α and cGAS-STING pathways. We have attempted to construct F317L-deficient ASFV (ASFVΔF317L), but we found that when the F317L gene was deleted in ASFV, the virus could not be rescued anymore. Therefore, we suspected that F317L was critical for ASFV replication and that complete deletion of F317L was lethal to the virus. Antivirals targeting F317L might be helpful for restricting ASFV replication.

TNF-α is one of the most pleiotropic proinflammatory cytokines, regulating a number of cellular responses, including cytotoxicity, antiviral activity, proliferation, and transcriptional regulation (35). TNF-α-induced NF-κB pathway activation plays a key role in antiviral immune response, and it shared downstream adaptors with cGAS-STING pathway (36). We determined that F317L inhibited TNF-α-induced inflammatory cytokine production and phosphorylation of IKKβ and IκBα. Therefore, we believed that the exact mechanism by which ASFV F317L inhibits NF-κB activation is that F317L interacts with IKKβ to prevent IKKβ phosphorylation and activation, thereby inhibiting the phosphorylation of IκBα and increasing the expression of IκBα. The upregulation of IκBα contributed to impaired inflammatory cytokine production and antiviral response. This mechanism is similar to vaccinia virus protein B14-mediated antagonistic function against NF-κB pathway activation (32).

**FIG 9:**
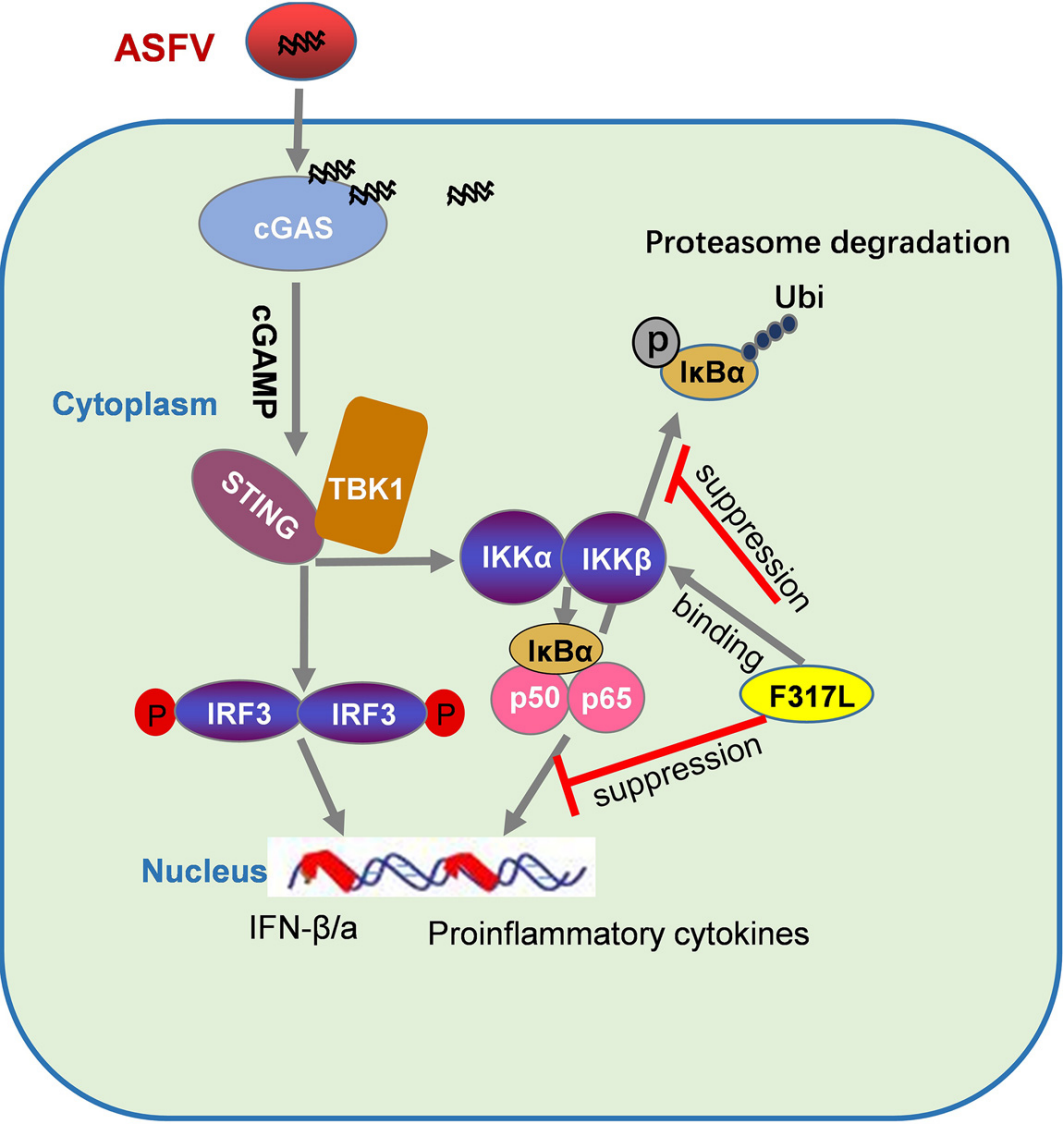
Diagram displaying the suppressive role of ASFV F317L protein on NF-κB pathway activation. F317L binds with IKKβ and inhibits the phosphorylation of IκBα, which decreases IκBα degradation by the proteasome and, in turn, blocks p65 nuclear translocation, inhibiting the expression of proinflammatory cytokines.

Phosphorylation of the IKKs is central to NF-κB activation. *trans*-Autophosphorylation of IKKs is decisive for IKK activation (37). Whether F317L interacts with the crucial phosphorylation sites in IKKβ or directly blocks the autophosphorylation of IKKβ remains unknown. However, this contributed to the decreased ubiquitination of IκBα, which stabilized IκBα and blocked NF-κB activation. The interaction domains or sites in IKKβ by F317L should be further investigated in the future to clarify the regulatory manner used by F317L. In summary, as depicted in Fig. 9, our study identified that F317L antagonizes NF-κB pathway activation by inhibiting NF-κB activation; therefore, F317L is a novel viral factor involved in innate immune evasion during ASFV infection. This provides a new target for development of ASFV recombinant live-attenuated vaccine or antivirals.

## MATERIALS AND METHODS

### Cell culture, viruses, and transfection.

Human embryonic kidney 293T (HEK-293T) and African green monkey fetal kidney cells (MA104) were cultured in Dulbecco’s modified Eagle medium (DMEM) containing 10% (vol/vol) fetal bovine serum (FBS; Gibco). Primary swine macrophages (PAM) and bone marrow-derived macrophages (BMDM) were cultured in RPMI 1640 medium supplemented with 20% (vol/vol) FBS. iPAM cells were cultured in RPMI 1640 medium supplemented with 10% (vol/vol) FBS; all the cells were cultured under 5% CO_2_ at 37°C. The ASFV CN/GS/2018 strain was isolated and stored in our lab previously (38). Transient transfection was performed with the desired plasmids using transfection reagents (jetPRIME) at a ratio of 2:1 (jetPRIME/DNA).

### Plasmids and antibodies.

The F317L gene of ASFV was amplified and cloned into pCMV-Flag4 vector using BamHI and EcoRI sites (Flag-F317L-expressing plasmid). The amplified coding sequence (CDS) was also cloned into pcDNA3.1-myc-his(-)A vector using EcoRI and BamHI sites (Myc-F317L-expressing plasmid). The IFN-β promoter luciferase reporter plasmid (constructed by inserting 3×IFN-β promoter into the pGL3-Basic vector) and other reporter plasmids, including NF-κB (NF-κB promoter driving firefly luciferase), pRL-TK (*Renilla* luciferase for internal control), ISRE luciferase reporter plasmids (ISRE element driving firefly luciferase), and the eukaryotic expressing plasmids expressing the indicated components of cGAS-SING pathway, including pRK-HA-cGAS, pRK-HA-STING, pRK-HA-TBK1, pRK-HA-IRF3, pRK-HA-IκBα, pRK-HA-IKKβ, and pRK-HA-p65, were kindly provided by Hongbing Shu (Wuhan University, China). The commercial antibodies used in this study include anti-Flag monoclonal antibody (Sigma-Aldrich), anti-HA tag antibody (Thermo Fisher Scientific), anti-β-actin monoclonal antibody (Santa Cruz Biotechnology), anti-p-IκBα-monoclonal antibody (Cell Signaling Technology), and anti-IκBα monoclonal antibody (Cell Signaling Technology), goat anti-mouse or rabbit IgG (H+L) secondary antibodies (Thermo Fisher Scientific), goat anti-mouse IgG heavy or light chain-specific secondary antibodies (Abbkine Scientific Co.), anti-p65 monoclonal antibody (Abcam), Alexa Fluor 488-conjugated IgG (H+L), and Alexa Fluor 594-conjugated IgG (H+L) antibodies (Thermo Fisher Scientific).

### Dual-luciferase reporter assay.

HEK-293T cells were cultured in 48-well plates. The monolayer cells were transfected with 50 ng of NF-κB-luc, ISRE-luc, or IFN-β-luc plasmid along with 5 ng/well of pRL-TK *Renilla* luciferase reporter plasmids and the indicated innate immune component-expressing plasmids (50 ng) and/or pCMV-Flag4-ASFV-F317L (50, 100, or 200 ng) plasmids. The empty vector plasmids were used in the whole transfection process to ensure that the cells received the same amounts of total plasmids. Cells were treated with the indicated stimuli and lysed at the proper time point with lysis buffer for 15 min at room temperature, and the luciferase activity was measured using a dual-luciferase assay kit according to the protocols provided by the manufacturer (Promega). All experiments were performed in triplicate and repeated at least three times.

### Co-IP and Western blotting.

HEK-293T cells were cultured in 10-cm tissue culture dishes, and the monolayer cells were cotransfected with the desired plasmids. The collected cells were lysed and immunoprecipitated with proper antibodies as described previously (39). For Western blotting, the samples were resolved by electrophoresis on a 10% SDS-PAGE gel and transferred to an Immobilon-P membrane (Millipore). The membrane was blocked using 5% skim milk in Tris-buffered saline with Tween 20 (TBST) and incubated with appropriate primary antibodies and secondary antibodies as described previously (40).

### qPCR assay.

Total RNAs were extracted using TRIzol reagent (Invitrogen), and 2 μg of total RNA of per sample was reverse transcribed into cDNA using PrimeScript RT master mix (TaKaRa). The Mx3005P quantitative PCR (qPCR) system (Agilent Technologies, Palo Alto, CA) and SYBR premix *Ex Taq* reagents (TaKaRa, Dalian, China) were used in the qPCR assay. The glyceraldehyde-3-phosphate dehydrogenase (GAPDH) gene was used as an internal control. The relative expression of mRNA was calculated based on the comparative cycle threshold (*C_T_*) (2^−ΔΔ^*^CT^*) method (41). ASFV genome copies were detected by absolute quantification assay as previously described (38). All experiments were repeated three times, with similar results. The data represent the results from one of the triplicate experiments. Primers are listed in Table 1.

**TABLE 1:** Primers used in this study

Gene	Sequence of primer (5′–3′)	
	Forward	Reverse
Human GAPDH	GAGTCAACGGATTTGGTCGT	GACAAGCTTCCCGTTCTCAG
Human IL-6	TTCTCCACAAGCGCCTTCGGTC	TCTGTGTGGGGCGGCTACATCT
Human IL-1β	CTGAGGAAGATGCTGGTTCCC	CAACACGCAGGACAGGTACAG
Human TNF-α	GCCGCATCGCCGTCTCCTAC	CCTCAGCCCCCTCTGGGGTC
Human IκB-α	TGCAGGCCACCAACTACAAT	AGCACCCAAAGACACCAACA
Porcine IL-6	CTGCTTCTGGTGATGGCTACTG	GGCATCACCTTTGGCATCTT
Porcine IL-1β	AAGATGACACGCCCACCCTG	TACCAGTTGGGGTACAGGGCA
Porcine TNF-α	GCCCAAGGACTCAGATCATC	GGCATTGGCATACCCACTCT
Porcine GAPDH	ACATGGCCTCCAAGGAGTAAGA	GATCGAGTTGGGGCTGTGACT
ASFV p30	GCAGGGCAAGGGTATACTGA	CTGTCTCCTCTTCAAACAGCAC
ASFV p72	CCGGGTACAATGGGTCTTCC	CGCAACGGATATGACTGGGA
ASFV F317L	ATGGTTGAGACACAAATGGA	CTATGTTGTGGACGATGCCTT

### RNA interference experiments.

The specific small interfering RNA (siRNA) designed for targeting ASFV F317L was synthesized by Genepharma (Shanghai, China). MA104 cells were transfected with negative control (NC) or F317L siRNAs. The target sequences of F317L were 5′-GCCGCAUUAAUAUGAUAAATTG-3′ (F317L-siRNA1) and 5′-GAUGCCUGCUGAUAUUUAUTT-3′ (F317L siRNA2). Nontargeting siRNA was used as a negative control (NC). At 6 h posttransfection (hpt), the cells were left uninfected or infected with ASFV at a multiplicity of infection (MOI) of 0.01. The mRNA expression levels of the target gene and viral indicators (ASFV p30 and p72 genes) were detected by qPCR.

### Indirect immunofluorescence assay (IFA).

HEK-293T cells were seeded into Nunc glass-bottom dishes and transfected with 1 μg of HA-IKKβ and 2 μg of Flag vector or Flag-F317L-expressing plasmids for 24 h. The collected cells were fixed with 4% paraformaldehyde in phosphate-buffered saline (PBS) at 4°C for 2 h. After 3 washings with ice-cold PBS, the cells were permeabilized with 0.2% Triton X-100 for 10 min. Bovine serum albumin (BSA; 5%) in PBS was used as the blocking buffer to prevent nonspecific background noise. After blocking at 37°C for 1 h, the cells were incubated with proper antibodies as described previously (42). The stained cells were examined with a Nikon Eclipse 80i fluorescence microscope.

### ELISA.

Cell culture supernatants were collected and the amounts of porcine IL-1β protein were assessed using a porcine IL-1β enzyme-linked immunosorbent assay (ELISA) kit (Solarbio) following the manufacturer’s instructions.

### Statistical analysis.

All data are presented as mean values ± standard errors (SE) from three independent experiments. Two-tail Student’s *t* test was used to analyze the significance of the data. Differences were considered statistically significant at a *P *value of <0.05, and a *P *value of <0.01 was considered highly significant.
